# Genetic Insights into Familial Hypospadias Identifying Rare Variants and Their Potential Role in Urethral Development

**DOI:** 10.3390/genes16111340

**Published:** 2025-11-06

**Authors:** Kholoud N. Al-Shafai, Seem Arar, Asma Jamil, Amina Azzah, Maraeh Mancha, Luis R. Saraiva, Tariq Abbas

**Affiliations:** 1 Division of Translational Medicine, Research Branch, Sidra Medicine, Doha P.O. Box 26999, Qatar; kalshafai@sidra.org (K.N.A.-S.); seemarar46@gmail.com (S.A.); ajamil@sidra.org (A.J.); aazzah-extern@sidra.org (A.A.); 2 Urology Division, Sidra Medicine, Doha P.O. Box 26999, Qatar; mmancha@sidra.org; 3 Department of Comparative Medicine, School of Medicine, Yale University, New Haven, CT 06510, USA; 4 College of Health and Life Sciences, Hamad Bin Khalifa University, Doha P.O. Box 34110, Qatar; 5 Weill Cornel Medicine-Qatar, Cornell University, Doha P.O. Box 24144, Qatar; 6 College of Medicine, Qatar University, Doha P.O. Box 2713, Qatar

**Keywords:** Hypospadias Biobank Cohort (HBC), familial hypospadias, whole genome sequencing, single nucleotide variants (SNVs) analysis, Qatar

## Abstract

**Background:** Hypospadias is a common congenital condition in male infants, characterised by incomplete development of the underside of the penile shaft. Genetic factors play a major role in its development. Therefore, studying genetic contributions, especially in familial cases, can enhance our understanding of disease causes and guide targeted interventions. **Materials and Methods:** Through a structured biobank for hypospadias, we collected blood samples from individuals with familial hypospadias and their relatives. Whole-genome sequencing (WGS) was performed on 27 individuals across seven families to identify potential genetic causes. Bioinformatics analysis, including the GEMINI tool, was used to assess inheritance patterns of single-nucleotide variants (SNVs) within families and identify potential causative SNVs. **Results:** We identified three likely pathogenic variants in genes not previously associated with hypospadias in *EIF2B5*, *INO80*, and *ACADVL* genes, in three index patients. These variants co-segregated with the condition within the families. Additionally, we detected variants of uncertain significance in hypospadias-related gene families (*DNAH12* and *LHFP*) and in other genes, such as *COL6A3*, which may cause the phenotype. No potential causative variants were found in two of the seven studied families, indicating the need for further analysis, including the assessment of copy number variants (CNVs). Functional studies will be crucial to establish the role of the identified variants in the development of hypospadias. **Conclusions:** This study underscores the importance of disease biobanking and genetic analysis in identifying potential underlying causes of congenital conditions, such as hypospadias. The identified variants provide new opportunities for functional research and may enhance our understanding of hypospadias pathophysiology. These findings broaden the genetic landscape of hypospadias and lay the groundwork for functional validation, improved risk assessment, and personalised medicine strategies.

## 1. Introduction

Hypospadias is among the most common congenital anomalies in males, occurring in about 2 out of every 1000 pregnancies, including live births, stillbirths, and terminations [1]. It is characterised by the ectopic placement of the urethral meatus on the ventral side of the penis, often accompanied by chordee and an abnormal foreskin. Despite surgical correction in early childhood, many individuals face long-term complications affecting urinary, reproductive, and sexual health [2]. Clinical management depends on accurate diagnosis and classification based on meatal location and severity, which guides the choice of surgical technique and helps predict outcomes [3].

While environmental and endocrine-disrupting exposures have been implicated, familial clustering and twin studies strongly support a genetic contribution to hypospadias, with heritability estimates ranging between 57% and 77% [4,5,6]. The development of male external genitalia critically depends on androgen signalling, particularly the actions of testosterone and its more potent derivative, dihydrotestosterone (DHT). Disruptions in this signalling axis caused by pathogenic variants in genes such as Androgen Receptor (*AR*) and Steroidogenic Factor 1 (*SF1*/*NR5A1*) can lead to incomplete virilisation and structural anomalies [7,8]. To date, at least 49 genes have been linked to hypospadias pathogenesis, covering androgen metabolism, hormone receptor activity, and urogenital development [9]. Furthermore, genome-wide association studies (GWASs) in sporadic hypospadias have identified several susceptibility loci containing developmental regulators including *HOXA4*, *IRX5*, and *EYA1* [4]. However, the genetic basis of hypospadias remains only partially understood, particularly in non-European populations.

To address this gap, we recently established the Hypospadias Biobank Cohort (HBC), which is a structured, longitudinal research platform integrating detailed clinical, phenotypic, and surgical data with biological specimens [10]. The HBC enables the systematic investigation of genotype–phenotype correlations and supports the refinement of classification frameworks for hypospadias severity and outcomes. As part of this effort, we recruited multiplex families with familial hypospadias and collected biospecimens from affected individuals and their relatives. In this study, we report whole genome sequencing (WGS) data analysis from 27 individuals across seven families within the HBC, aiming to identify rare or high-impact genetic variants that may underlie hypospadias pathogenesis in this understudied population, where inbreeding and consanguinity are common practices that would influence the genetic architecture of hypospadias, as noticed for other disease phenotypes [11].

## 2. Material and Methods

### 2.1. Ethical Approval

Ethical approval was obtained from the Institutional Review Board of Sidra Medicine (IRB #1866246), and the study was conducted in accordance with the Declaration of Helsinki. Informed consent/assent was obtained from all study participants to establish a structured Hypospadias Biobank Cohort (HBC) at Sidra Medicine in Qatar, for which comprehensive data and samples were collected from participants for research purposes, as detailed elsewhere [10].

Participants in the HBC were selected based on specified inclusion criteria to ensure broad representation across the spectrum of hypospadias, including distal, midshaft, and proximal forms [10]. Urethral defect ratio (UDR) was calculated by dividing the extent of the urethral defect (distance between the glandular knobs and BCS) by the stretched penile length (SPL). Hypospadias severity was then classified into three distinct grades (UDR < 0.5, 0.5–0.99, ≥1.0). Participants with additional syndromic conditions were excluded to reduce the complexity of genetic analyses, enabling a more focused examination of isolated hypospadias. Finally, participants from familial cases of hypospadias, where more than one family member has hypospadias, were considered eligible, as this suggests a potential genetic predisposition. Individuals with syndromic conditions or known genetic disorders that could interfere with future analyses were excluded, along with eligible participants who were unable to provide informed consent or assent due to language barriers or medical reasons.

### 2.2. Study Participants

**Figure 1:**
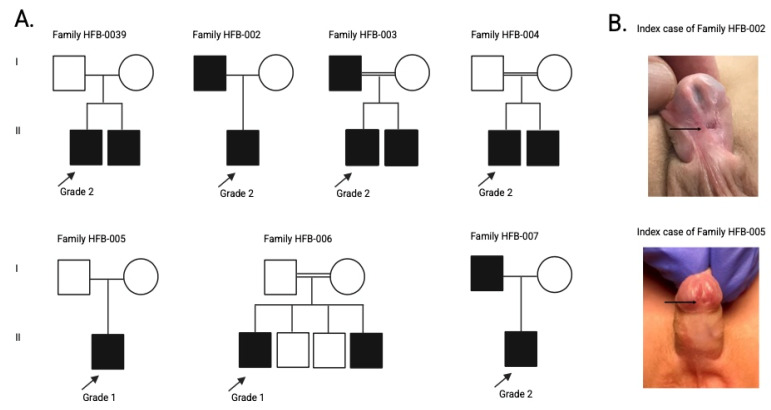
(**A**) Pedigrees of the seven families with hypospadias studied. quares represent males, circles represent females, filled symbols indicate affected individuals, and open symbols indicate unaffected individuals. A double horizontal line denotes consanguineous unions. Roman numerals (I, II) indicate family generations. The hypospadias grade for each index case is shown below the corresponding symbol. The fathers of the index cases of HFB-005 and HFB-006 are first cousins. (**B**) Clinical images of two index cases with different hypospadias phenotypes. The index case of Family HFB-002 had Grade 2 hypospadias, characterised by a higher urethral defect ratio, while the index case of Family HFB-005 had Grade 1 hypospadias, defined by a lower urethral defect ratio. The arrows indicate the level of the hypospadiac meatus opening in each case.

Seven familial cases of hypospadias and their relatives (total n = 27) were identified and enrolled at HBC for sample and data collection, followed by genetic investigation. Familial cases of hypospadias refer to situations in which more than one family member is affected by the same condition, suggesting a genetic or hereditary basis. The 27 study participants included 7 index cases and 20 of their family members (7 affected, 13 unaffected). Family pedigrees are provided in Figure 1.

### 2.3. Whole Genome Sequencing (WGS) Data Generation and Processing

A total of 4–10 mL of peripheral blood samples, collected into EDTA-containing tubes, were obtained from each participant for genomic DNA extraction, followed by WGS library preparation using the Truseq DNA Nano Kit (Illumina, San Diego, CA, USA) at the Sidra Integrative Services Laboratory. Sequencing was conducted on the NovaSeq 6000 platform (Illumina), achieving an average coverage of 30–40×. Our analysis pipeline involved demultiplexing the raw sequencing data (.bcl files) into FastQ files. Read quality was evaluated with FastQC (https://github.com/s-andrews/FastQC, accessed on 1 July 2024), and adapter sequences were trimmed using Trimadap (https://github.com/lh3/trimadap, accessed on 1 July 2024). High-quality reads were aligned to the human reference genome GRCh37/hg19 (NIH, New York, NY, USA) with BWA-MEM from Burrows-Wheeler Aligner v7.0.8 (Cambridge, MA, USA; arXiv:1303.3997 [q-bio.GN]). Duplicate reads were marked with SAMBLASTER v0.1.22, and alignment files were sorted using SAMtools from the BWA-kit package [12]. Picard and Mosdepth were utilised for additional quality control of the alignments. After base quality score recalibration, variants were called with GATK v4.0.9.0 [13]. Individual gVCF files were jointly called to produce a merged VCF file. These joint VCFs were split, decomposed, and normalised using Variant Transformer v0.57 [14]. Variants were annotated with SnpEff v4.3 [15], allele frequency databases such as gnomwAD [16] and the Greater Middle Eastern [17], clinical databases like ClinVar [18], and pathogenicity prediction scores including SIFT [19], PolyPhen-2 [20], and CADD [21].

### 2.4. Single-Nucleotide Variant (SNV) Segregation Analysis and Filtration

**Figure 2:**
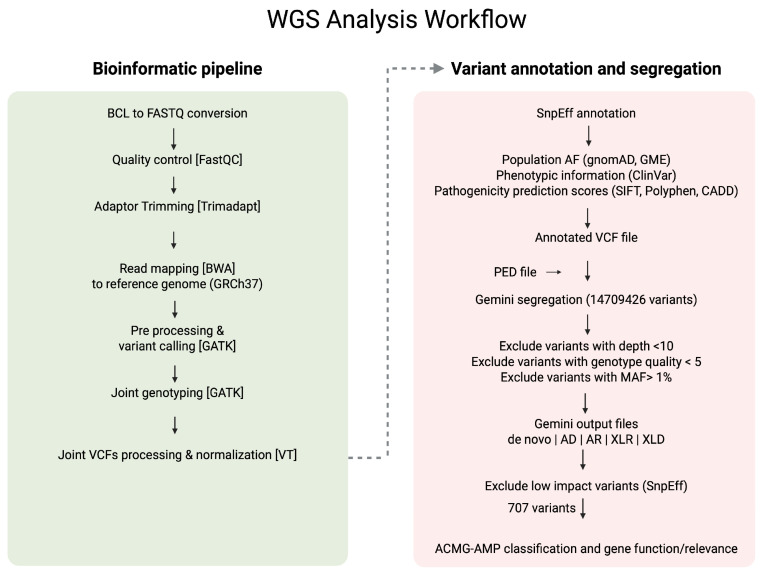
Workflow of the whole genome sequencing (WGS) analysis used in the study.

Segregation analysis of single nucleotide variants (SNVSs) was performed using Gemini v0.30.2 [22]. The annotated VCF files, along with corresponding pedigree information, were imported into the Gemini platform using the vcf2db utility. This facilitated the exploration of various inheritance models, including de novo, autosomal dominant, autosomal recessive, compound heterozygous, X-linked recessive, and X-linked dominant patterns. A custom Python v.3.9.0 script was employed to export Gemini query results into Excel spreadsheets for further filtering and interpretation. Each family was analysed based on the potential mode of inheritance suggested by the pedigree information. Variants were excluded if they had a read depth less than10, genotype quality less than 5, a minor allele frequency (MAF) above 1% in population databases, or were predicted to have low functional impact according to SnpEff annotations. The remaining variants were subsequently evaluated and classified following the ACMG-AMP guidelines [23] using Varsome [24] and Franklin (https://franklin.genoox.com/clinical-db/home, accessed on 1 July 2024), with a literature review and gene function assessment. Figure 2 provides a clear illustration of the variant filtering and analysis workflow that was used in the study.

## 3. Results

### 3.1. Demographic and Clinical Characteristics

For this study, a total of 27 individuals were enrolled across the seven families, comprising the seven index cases and 20 relatives (7 affected, 13 unaffected), to enable combined clinical and genetic analyses (Figure 1). The demographic and clinical features of the seven index cases with familial hypospadias enrolled in this study are summarised in Table 1. The median age at diagnosis was 3 years (range: 2–10 years). All index cases were male, and the cohort encompassed a diverse set of ethnic backgrounds, including Sudanese, Indian, Pakistani, Palestinian, Yemeni, and Egyptian nationalities. Five of the seven probands (71%) were of Arab ethnicity, reflecting the population structure. Parental consanguinity was reported in three families, consistent with the high consanguinity rates observed in the Middle Eastern countries [25] and Pakistan [26]. All index cases had a positive family history of hypospadias, supporting a likely genetic aetiology. Treatment included surgical correction in all cases, with two patients also receiving preoperative intramuscular testosterone injections of 2 mg/kg twice monthly.

### 3.2. Variants Detected from WGS Analysis

WGS of the seven families with familial hypospadias described above identified multiple rare variants, some with potential pathogenic relevance (Figure 2 and Table 2). Supplementary Figure S1 provides the Integrated Genomics Viewer (IGV) screenshots of the described variants, in the index cases and relatives. Two of these variants were classified as pathogenic (P) and three as likely pathogenic (LP), along with four variants of uncertain significance (VUS), some of which may be pathogenic and are located in genes not previously linked to hypospadias aetiology.

### 3.3. Pathogenic and Likely Pathogenic Gene Variants

Three out of the seven families had at least one pathogenic or likely pathogenic variant identified. In Family HFB-002, a pathogenic splice site gene variant (c.642+1G>A) and a likely pathogenic missense variant (c.821G>A) were identified in *TTC37* and *EIF2B5* genes, respectively. These variants were inherited from the affected father in a heterozygous state. The TTC37-encoded protein is involved in RNA degradation activities [27]. Mutations in the *TTC37* gene have been linked to Trichohepatoenteric syndrome (syndromic diarrhoea) in both homozygosity and compound heterozygosity [28]. The variant has a high SpliceAI score (0.94), suggesting it would influence splicing activity [29]. The protein encoded by *EIF2B5* is critical for recycling GDP-bound eIF2 into its active GTP-bound form, an essential step in initiating protein synthesis. Mutations in *EIF2B5* impair the regulation of protein synthesis during cellular stress, leading to vanishing white matter disease, a progressive leukodystrophy characterised by neurological deterioration [30]. A VUS was also detected in this family within the *DNAH12* gene, as described later in this manuscript.

In Family HFB-003, a heterozygous frameshift variant (c.458dupG) in the *OBSL1* gene, classified as pathogenic, was identified. *OBSL1* encodes obscurin-like protein 1, a cytoskeletal protein involved in muscle structure and function. A recent study demonstrated that a homozygous *OBSL1* mutation (c.848delG) can cause Three M Syndrome 2 (3M2), a rare growth disorder characterised by short stature and distinctive facial and skeletal features, in a Pakistani family. Interestingly, affected members of that family also exhibited atypical features, including hypospadias [31]. Furthermore, a heterozygous missense variant (c.445G>C) in the *INO80* gene, classified as likely pathogenic, was also detected in Family HFB-003. *INO80* encodes a chromatin remodelling factor that regulates Bone Morphogenetic Protein 4 (BMP4) expression, a well-established gene involved in urethral development and hypospadias pathogenesis [32,33].

In Family HFB-007, a likely pathogenic heterozygous variant (c.67C>T) was identified in *ACADVL*, both of which co-segregated with the phenotype within the family. The *ACADVL* gene encodes the enzyme very long-chain acyl-CoA dehydrogenase, which is vital for the mitochondrial β-oxidation of very long-chain fatty acids. Mutations in *ACADVL* cause the autosomal recessive disorder very long-chain acyl-CoA dehydrogenase deficiency (VLCADD; OMIM #201475), which can manifest as early onset cardiomyopathy, hypoketotic hypoglycaemia, or adult-onset rhabdomyolysis.

### 3.4. Variants of Uncertain Significance (VUS)

Two homozygous VUS were identified in two of the studied families (Family HFB-0039 and Family HFB-004). In Family HFB-0039, a rare homozygous splice site variant (c.-176A>G) in the *LHFP* gene was identified in the two affected males of one family, while the unaffected parents were heterozygous for this variant. Although the *LHFP* gene itself has not been previously linked to hypospadias, functional studies of related gene family members such as *LHFPL2* have reported causes of hypospadias-like phenotypes in mice and suggested a mechanistic role of the encoded protein in genital development [34]. Moreover, in Family HFB-004, a homozygous missense variant (c.3223C>T) of uncertain significance (VUS) was identified in *COL6A3*, a gene implicated in extracellular matrix integrity but not previously linked to genital malformations. However, *COL6A3* gene expression was upregulated in *DNAH17* mutants detected in severe hypospadias [7] and therefore requires further assessment.

In addition to these two homozygous variants, a heterozygous missense VUS variant (c.7604C>T) in *DNAH12* was identified in Family HFB-002, in both the index case and his affected father. The *DNAH12* gene encodes a dynein axonemal heavy chain, a motor protein involved in microtubule-based movement and ciliary function, including sperm motility. Although rare damaging variants in several dynein heavy chain genes have been linked to hypospadias, *DNAH12* itself has not been specifically implicated, and a recent study found no enrichment of *DNAH12* mutations in severe hypospadias cases compared with the controls [7]. Interestingly, a recent study reported that male mice lacking Dnah12 were infertile and displayed smaller testes with reduced sperm counts, whereas females developed normally [35].

No candidate variants were identified in two related families (Family HFB-005 and Family HFB-006), suggesting the need for further investigation into non-coding variants, regulatory elements, or structural variation such as copy number variants (CNVs). The absence of non-coding region and CNV analyses represents a limitation of this study, as regulatory or structural variants may underlie the observed phenotypes. Future functional validation experiments will be essential to confirm the pathogenicity of the prioritised variants and to establish mechanistic links to hypospadias development.

## 4. Discussion

The findings from this study offer important insights into the genetic foundations of familial hypospadias and its potential hereditary causes in highly inbred and understudied populations. In this cohort of seven families with familial hypospadias, we identified several rare variants with likely pathogenic significance, located in genes that have not previously been linked to the hypospadias phenotype. These results not only add to the growing body of knowledge on the genetic basis of hypospadias but also emphasise the complexity and diversity of its aetiology, potentially involving both common and rare genetic variants that influence urethral and genital development.

The *INO80* missense variant (c.445G>C) also seems promising, as INO80 is involved in chromatin remodelling and regulates BMP4, a key gene in urethral development [32,33]. The role of chromatin remodelling factors in hypospadias pathogenesis has not been extensively studied, making this a potentially novel and important pathway to explore in future research. The same family also has the *OBSL1* frameshift variant, with previous evidence suggesting that mutations in this gene can be associated with the hypospadias phenotype [31].

Among the identified variants, three VUS variants stood out. This includes the homozygous *LHFP* splice site mutation found in family HFB-0039, which is especially noteworthy. Although *LHFP* has not previously been linked to hypospadias, functional studies on related genes (*LHFPL2*) suggest a role in genital development [34]. Specifically, *LHFPL2* mutant mice exhibit elongation of Müllerian and Wolffian ducts; however, their duct tips are enlarged and fail to merge with the urogenital sinus, emphasising the importance of this gene in distal reproductive tract development [34]. This finding raises the possibility that mutations in *LHFP,* such as the identified variant c.-176A>G, may contribute to the development of hypospadias, warranting further investigation. Additionally, the identification of another homozygous variant of uncertain significance (VUS) in *COL6A3*, a gene previously implicated in extracellular matrix integrity, highlights a potential new area of interest warranting further investigation. Notably, *COL6A3* expression has been reported to be upregulated in *DNAH17* mutants associated with severe hypospadias [7], suggesting a possible link that merits closer examination. Notably, neither the *LHFP* nor the *COL6A3* variants were detected as homozygous in gnomAD v4.1.0 [36]. Also, a heterozygous missense VUS (c.7604C>T) in *DNAH12* was identified in both the index case and his affected father in Family HFB-002. Although *DNAH12* has not been directly implicated in hypospadias, its role in ciliary function and sperm motility suggests potential relevance to male reproductive development. Notably, animal studies have shown that male *Dnah12*-knockout mice are infertile and exhibit testicular abnormalities, supporting a possible role in testis or sperm function [35]. However, current human data do not indicate a significant enrichment of *DNAH12* variants in hypospadias cases [7]. Therefore, while this variant remains of uncertain significance, its biological function warrants further investigation, particularly in the context of ureteral development.

Although these findings offer valuable insights, the identification of VUS in *LHFP*, *COL6A3*, and *DNAH12* highlights the importance of caution when interpreting the clinical relevance of these genetic changes, and further validation is needed to establish their pathogenicity.

Our results underscore the necessity for ongoing research into non-coding variants, regulatory elements, and structural variations, including CNVs [37], which might contribute to the development of hypospadias, especially in the two related families (Family HFB-005 and Family HFB-006) for which no candidate variants were identified.

From a clinical perspective, identifying genetic variants linked to familial hypospadias has the potential to significantly improve our understanding of the condition’s pathogenesis, diagnosis, and management [38]. Currently, hypospadias is diagnosed through clinical examination, and treatment mainly involves surgical correction [6]. However, increasing recognition of a genetic component in hypospadias suggests that genetic testing could become an essential diagnostic tool, especially in familial cases. Identifying specific genetic mutations could not only facilitate early diagnosis but also offer valuable insights for predicting severity and guiding treatment choices. For example, finding genetic variants associated with more severe forms of hypospadias could help clinicians customise surgical approaches based on the patient’s genetic profile. Additionally, genetic insights may enable the identification of individuals at higher risk in future pregnancies, aiding prenatal counselling and testing. Genetic testing could also help distinguish isolated hypospadias from other congenital anomalies with similar phenotypes, thereby improving clinical accuracy. Furthermore, these genetic discoveries might contribute to the development of targeted therapies, particularly when surgical correction alone is inadequate or when the condition is linked to significant comorbidities. For instance, if genes such as *INO80* and *BMP4* are involved in the pathogenesis of hypospadias, therapeutic strategies that target chromatin remodelling or *BMP4* signalling could be explored [32,33].

While the findings from this study are promising, several avenues for future research will be essential for advancing our understanding of familial hypospadias. First, larger cohort studies, including both familial and sporadic cases, are necessary to confirm the association of the identified variants with hypospadias. Since the genetic architecture of hypospadias is likely complex, involving multiple genetic factors, further research should aim to identify additional genes and variants that may contribute to its development. Future investigations should also expand the scope of genetic analysis to encompass non-coding regions of the genome and structural variations, such as CNVs [37]. These regulatory elements and large genomic rearrangements might play a role in hypospadias development and remain largely unexplored. Additionally, functional validation studies are required to verify the pathogenicity of the identified variants and understand their molecular mechanisms. Techniques like CRISPR-Cas9-based gene editing in model organisms could help explore the functional effects of these variants on genital development. An important direction for future research is to develop improved tools for genetic counselling and prenatal screening for hypospadias. As more genetic markers are identified, creating reliable and cost-effective genetic tests suitable for clinical use will be crucial. Moreover, discovering genetic risk factors can enhance predictive models for assessing the recurrence risk of hypospadias within families, aiding family planning and early intervention. Ultimately, a multidisciplinary approach involving paediatric urologists, geneticists, and functional biologists will be vital to translating these genetic findings into clinical practice. By fostering collaboration across specialities, we can ensure that insights from genetic research are effectively applied to improve care and outcomes for individuals with hypospadias.

Although the present study focused on familial cases and genetic findings, hypospadias is widely recognised as a multifactorial condition resulting from a complex interplay between genetic susceptibility and environmental exposures. Several studies have reported associations between maternal exposure to endocrine-disrupting chemicals, hormonal treatments during pregnancy, and increased risk of hypospadias [39]. Moreover, emerging evidence suggests that epigenetic mechanisms, including aberrant DNA methylation and histone modifications in developmental or hormone receptor genes, may influence urethral formation [40]. These findings underscore the potential importance of gene–environment interactions and epigenetic regulation in the aetiology of hypospadias, warranting further integrative studies to disentangle their relative contributions

## 5. Conclusions

This study highlights the critical role of disease biobanking and comprehensive genetic analysis in uncovering the potential underlying causes of congenital conditions such as hypospadias. By leveraging large-scale genomic data, we were able to identify specific genetic variants that may contribute to the development of this condition, offering new insights into its complex aetiology. These findings not only open avenues for future functional investigations to validate the biological relevance of these variants but also pave the way for the development of more targeted diagnostic tools and potential therapeutic strategies. Furthermore, this research underscores the broader value of integrating biobank resources with advanced genomic technologies to better understand rare and understudied congenital anomalies. Ultimately, deepening our understanding of the genetic architecture of hypospadias in understudied populations holds promise for improving early detection, personalised care, and long-term outcomes for affected individuals in this region and beyond.

## Figures and Tables

**Table 1:** Basic and clinical features of the index cases (I) of the studied families (n = 7) with hypospadias.

Feature	HFB-0039-I	HFB-002-I	HFB-003-I	HFB-004-I	HFB-005-I	HFB-006-I	HFB-007-I
**Age**	3 years	2 years	2 years	6 years	3 years	7 years	10 years
**Nationality**	Sudanese	Indian	Pakistani	Palestinian	Yemeni	Yemeni	Egyptian
**Consanguinity** **(Yes/No/Not reported)**	No	No	yes	yes	Not reported	Yes	No
**Grade of Hypospadias**	2	2	2	2	1	1	2
**Treatment Given (Surgery/Medication/None)**	Surgery/ Testosterone	Surgery	Surgery	Surgery	Surgery	Surgery	Surgery/ Testosterone

**Table 2:** Summary of genetic findings of the SNV analysis of the studied families (n = 7).

Family ID	Gene	Nucleotide Change	AA Change	Variant Impact	RS ID	GnomAD Genome AF (v4.1.0)	CADD Score	ACMG/AMP Classification	Zygosity	Variant Inheritance in Our Families
**HFB-0039**	*LHFP*	c.-176A>G	-	Splice region	rs866455435	0.0002036	9.2	VUS	Hom	Inherited from unaffected het. parents Affected brother also homozygous
**HFB-002**	*TTC37*	c.642+1G>A	-	Splice donor	rs538894487	0.00001313	34	P	Hett	Inherited from affected het. father
	*EIF2B5*	c.821G>A	p.Arg274Gln	Missense	rs1460067929	0.000006574	33	LP	Het	Inherited from affected het. father
	*DNAH12*	c.7604C>T	p.Pro2535Leu	Missense	rs529341489	0.00006570	31	VUS	Het	Inherited from affected het. father
**HFB-003**	*OBSL1*	c.458dupG	p.Leu154fs	Frameshift	rs767237510	0.00003959	-	P	Het	Inherited from affected het. father Affected brother also heterozygous
	*INO80*	c.445G>C	p.Glu149Gln	Missense	rs749039242	0.00001315	18.9	LP	Het	Inherited from affected het. father Affected brother also heterozygous
**HFB-004**	*COL6A3*	c.3223C>T	p.Arg1075Trp	Missense	rs201962257	0.0004079	25.6	VUS	Hom	Inherited from unaffected het. parents Affected brother also homozygous
**HFB-005**	-	-					-			
**HFB-006**	-	-					-			
**HFB-007**	*ACADVL*	c.67C>T	p.Gln23 *	Stop gain	rs1237915800	0.00000658	24.9	LP	Het	Inherited from affected het. father

AA = amino acid; AF = allele frequency; Het = heterozygous; Hom = homozygous; VUS = variant of uncertain significance; LP = likely pathogenic; P = pathogenic; * = frameshift. Allele frequency data from gnomAD v4.1.0 (based on GRCh38) were lifted over to the GRCh37/hg19 reference build using the Broad Institute’s LiftOver tool (https://liftover.broadinstitute.org) to ensure compatibility with the reference genome used for alignment and variant annotation.

## Data Availability

The datasets generated and analysed during the current study are not publicly available due to participant confidentiality and institutional data-sharing policies. However, de-identified data may be made available upon reasonable request to the corresponding author, subject to approval from the relevant ethics committee and compliance with institutional regulations.

## Supplementary Materials

The following supporting information can be downloaded at: https://www.mdpi.com/article/10.3390/genes16111340/s1, Figure S1: Integrated Genomics Viewer (IGV) screenshots of the described variants in index cases and relatives.
