# Supplementary material for: Genetic Insights into Familial Hypospadias Identifying Rare Variants and Their Potential Role in Urethral Development
Source: Genes (Basel). 2025 Nov 6;16(11):1340. doi: 10.3390/genes16111340 (PMC12652443; doi:10.3390/genes16111340)

Supplementary Figure 1: Integrated Genomics Viewer (IGV) screenshots of the described variants in index cases and relatives.

- *LHFP* gene variant
  - Chromosome 13, position 40177020–40177021
  - Index patient: 0201360010
  - Relatives: 0201384160 (mother), 0201384150 (unaffected father), 0201384170 (affected brother)

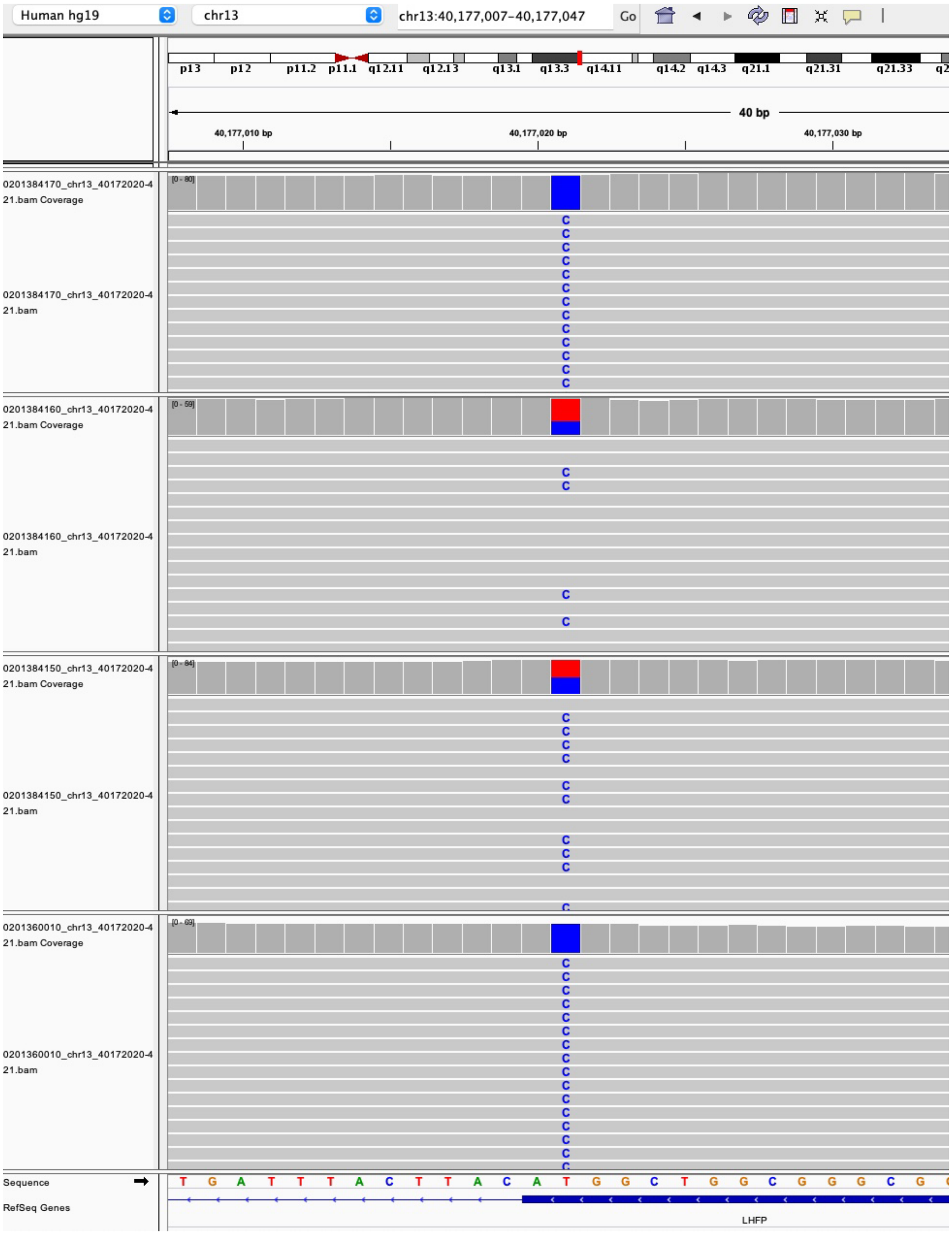

- *TTC37* **gene variant**
    - Chromosome 5, position 94872745–94872746
    - Index patient: 0201402070
    - Relatives: 0201384810 (mother), 0201384800 (affected father)

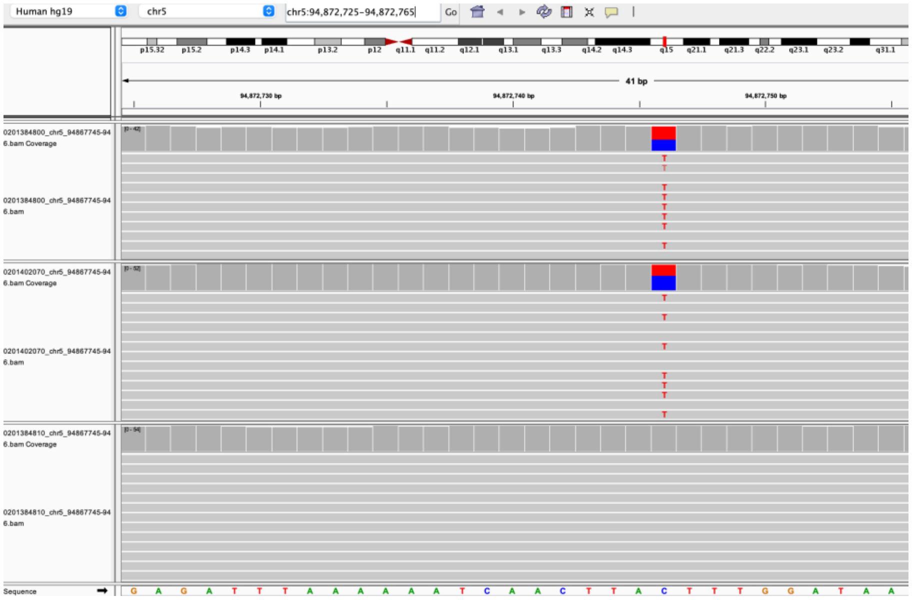

- *EIF2B5* **gene variant**
    - Chromosome 3, position 183857922–183857923
    - Index patient: 0201402070
    - Relatives: 0201384810 (mother), 0201384800 (affected father)

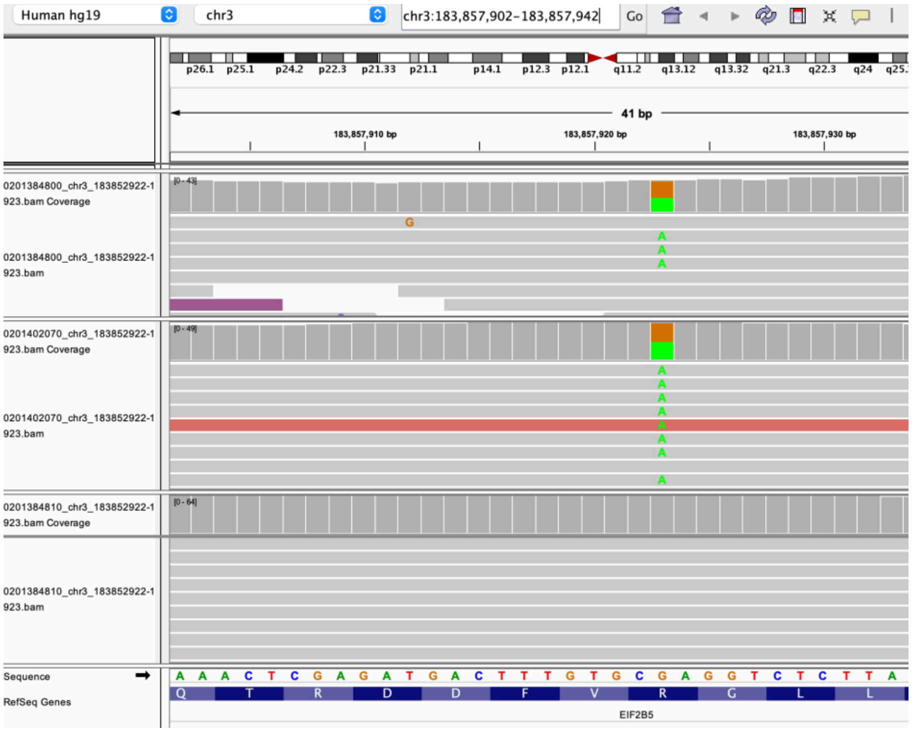

- *DNAH12* **gene variant**
    - Chromosome 3, position 57357209–57357210
    - Index patient: 0201402070
    - Relatives: 0201384810 (mother), 0201384800 (affected father)

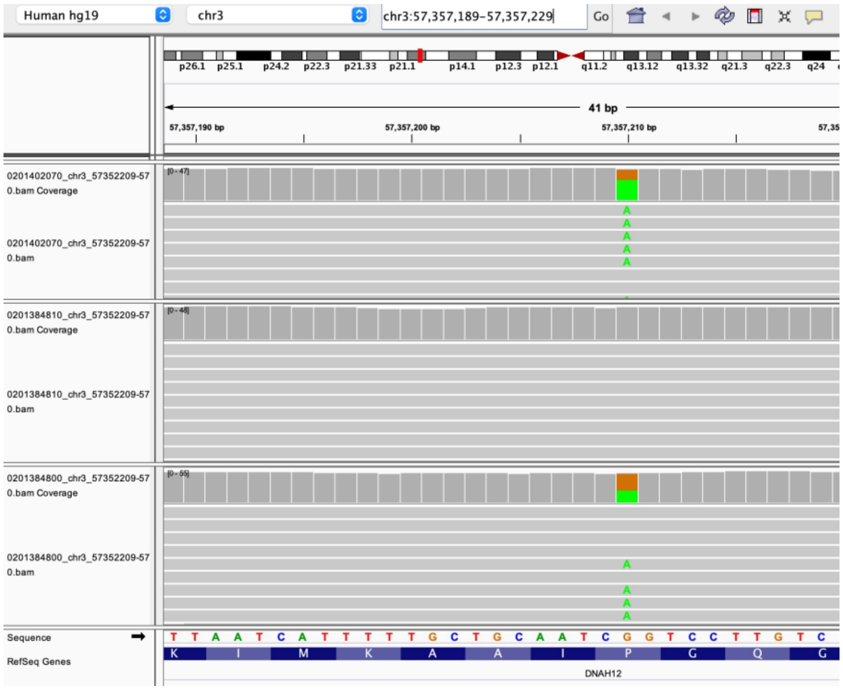

- *OBSL1* **gene variant**
    - Chromosome 2, position 220435495–220435496
    - Index patient: 0201402080
    - Relatives: 0201385190 (mother), 0201385180 (affected father), 0201385200 (affected brother)

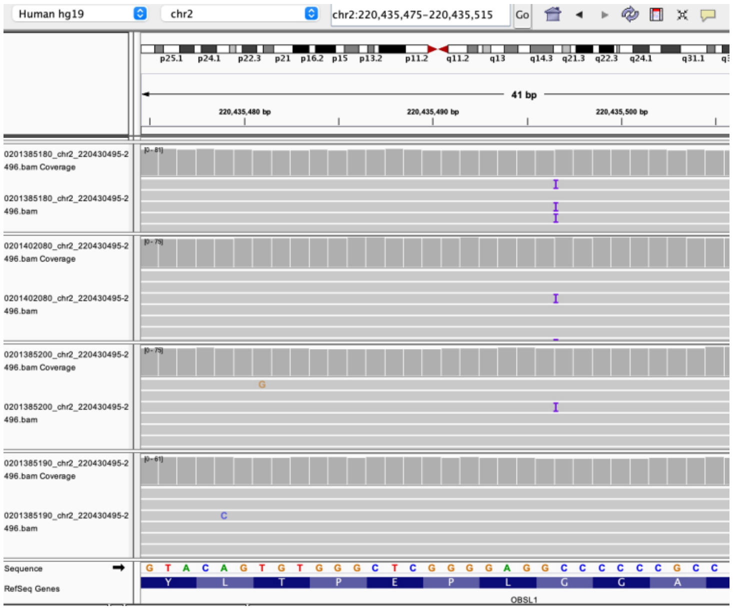

- ***INO80* gene variant**
    - Chromosome 15, position 41384316–41384317
    - Index patient: 0201402080
    - Relatives: 0201385190 (mother), 0201385180 (affected father), 0201385200 (affected brother)

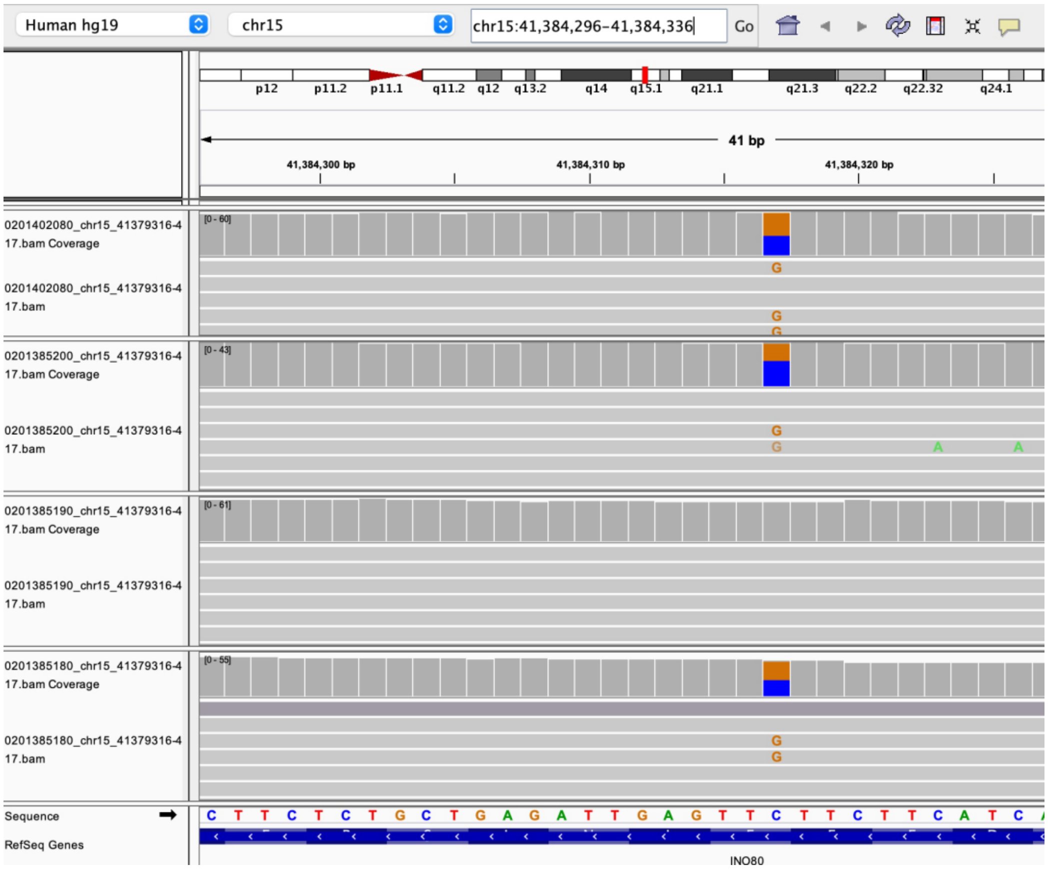

- *COL6A3* **gene variant**
    - Chromosome 2, position 238283510–238283511
    - Index patient: 0201402030
    - Relatives: 0201402050 (mother), 0201402040 (unaffected father), 0201402060 (affected brother)

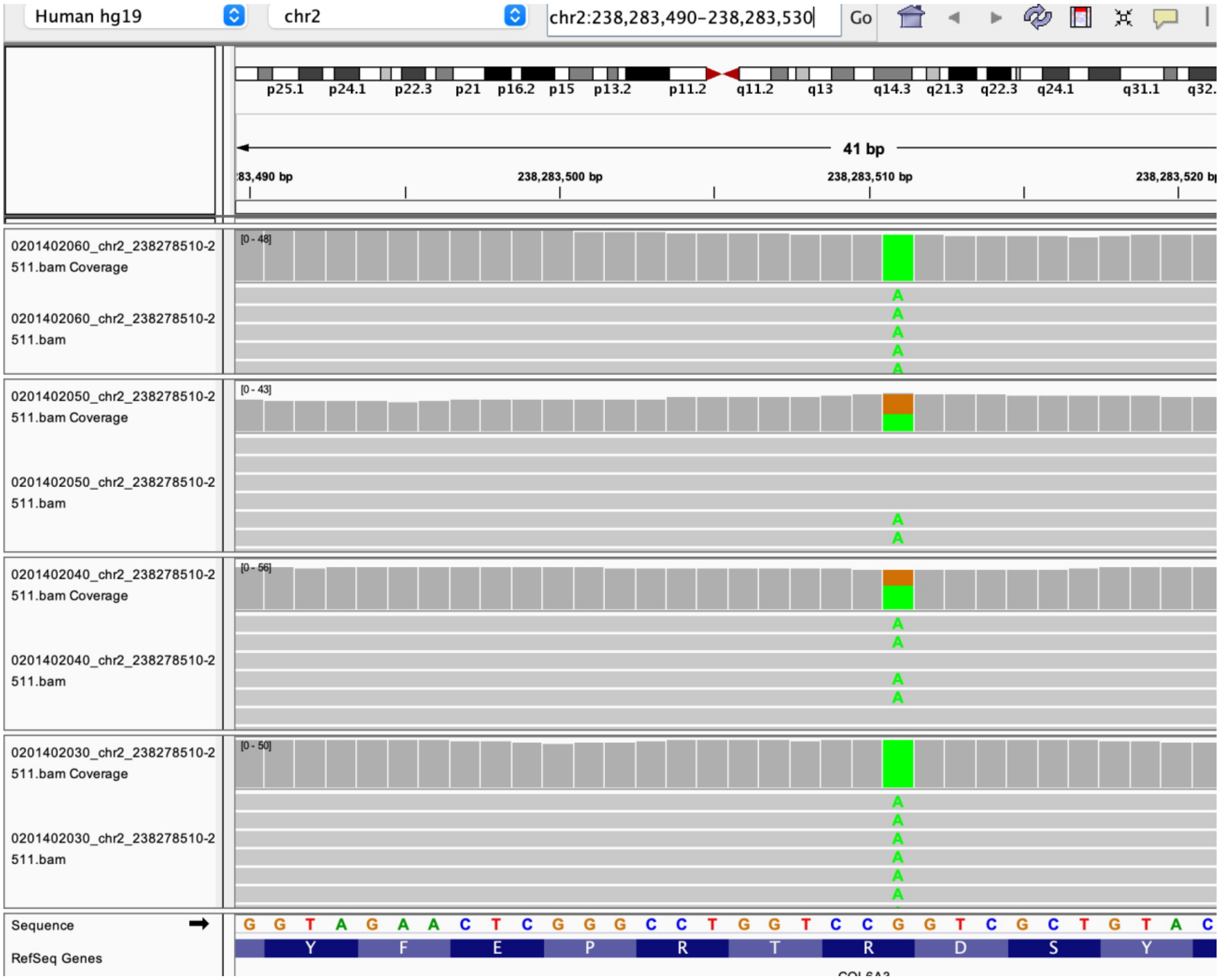

- *ACADVL* **gene variant**
  - Chromosome 17, position 7121072–7121073
  - Index patient: 0201408840
  - Relatives: 0201408820 (mother), 0201408810 (affected father)

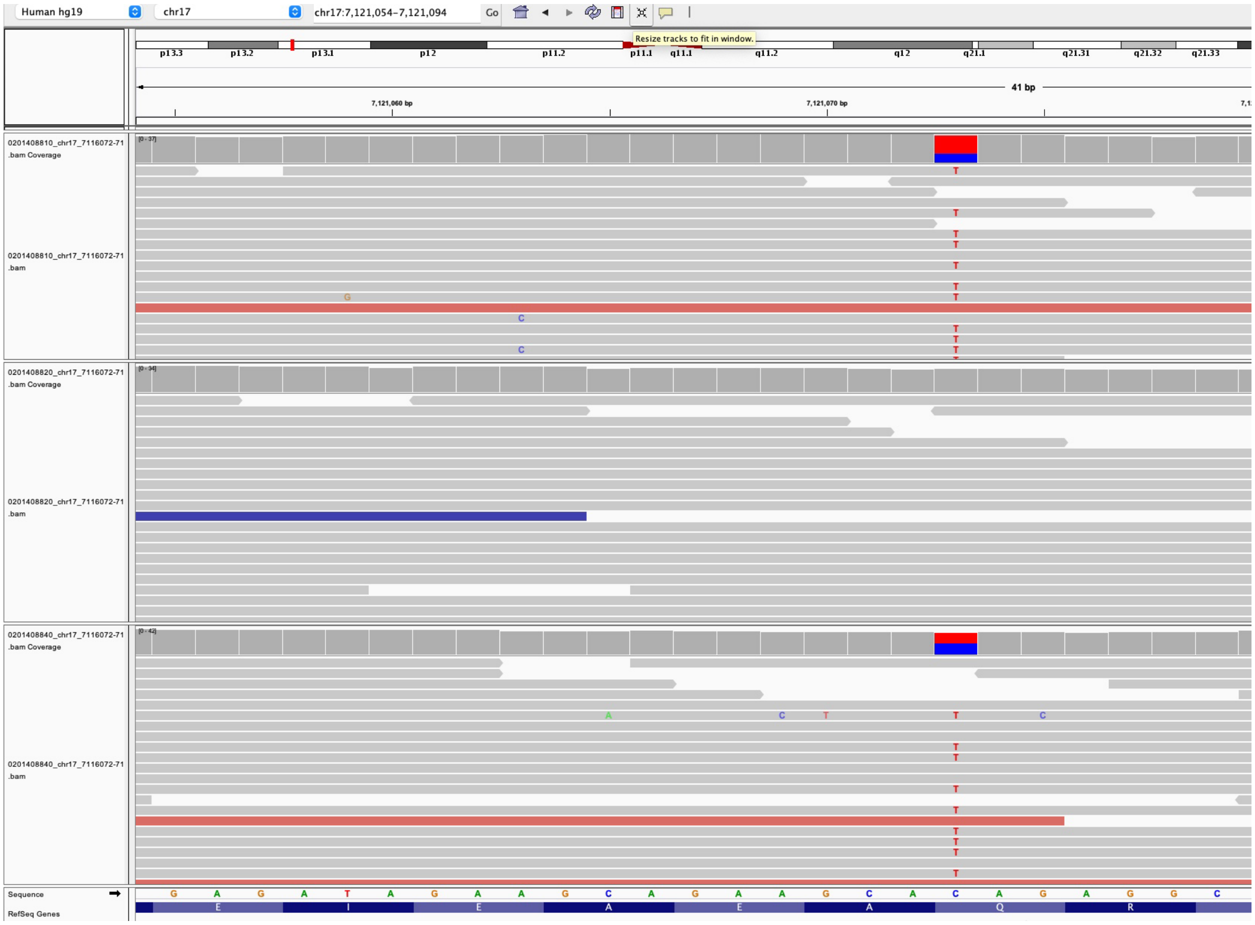

Supplement: Supplementary file 1 [file genes-16-01340-s001.zip › genes-3961471-supplementary.pdf]
